# Knowledge, attitudes, and practices regarding hyperuricemia among physicians in internal medicine departments: a multicenter cross-sectional survey in China

**DOI:** 10.3389/fpubh.2026.1750197

**Published:** 2026-05-18

**Authors:** Xiaofeng Chen, Chongling Zhang, Xiaoqin Li, Mei Yang, Lijie Liu, Xuelan Chen, Haiping Yang

**Affiliations:** Ministry of Education Key Laboratory of Child Development and Disorders, Chongqing Key Laboratory of Pediatrics, Chongqing Key Laboratory of Pediatric Metabolism and Inflammatory Diseases, Department of Nephrology, National Clinical Research Center for Child Health and Disorders, Children’s Hospital of Chongqing Medical University, Chongqing, China

**Keywords:** knowledge, attitude, practice, hyperuricemia, physicians, internal medicine

## Abstract

**Introduction:**

Hyperuricemia (HUA) is a highly prevalent metabolic disorder associated with gout and multiple cardiometabolic and renal comorbidities, making its management important in internal medicine practice. This study aimed to assess the knowledge, attitudes, and practices (KAP) of internal medicine physicians concerning HUA.

**Methods:**

This study implemented a multicenter observational study spanning from November 2024 to March 2025, encompassing 25 healthcare facilities. Information was obtained through a comprehensive survey instrument adapted from prior research and relevant guidelines and pilot-tested for reliability that captured participant demographics and assessed HUA-related KAP metrics. Cutoff values for KAP scores were determined based on Bloom’s criteria: scores of 80–100% were considered good, 60–79% moderate, and below 60% poor.

**Results:**

The analysis incorporated 390 complete survey responses, achieving an effective completion rate of 96.30%. Participant demographics revealed a predominantly female cohort (84.62%), averaging 36.02 ± 6.91 years of age. Most respondents were affiliated with tertiary hospitals (87.44%) and institutions with teaching responsibilities (89.74%). Mean scores across the three domains were: knowledge 5.51 ± 2.04 (potential range: 0–12), attitude 41.67 ± 3.59 (potential range: 9–45), and practice 40.98 ± 8.45 (potential range: 10–50), corresponding to poor knowledge (<60% of maximum), good attitude (≥80%), and moderate practice (60–79%) based on Bloom’s criteria. Correlation analysis showed that both knowledge (*r* = 0.1492, *p* = 0.0031) and attitude scores (*r* = 0.5369, *p* < 0.001) were positively correlated with practice scores. Structural equation modeling indicated significant direct effects of knowledge (*β* = 0.84, *p* < 0.001) and attitude (*β* = 1.05, *p* < 0.001) on practice.

**Conclusion:**

Despite generally positive attitudes and moderate practices toward HUA management, internal medicine physicians demonstrated suboptimal levels of knowledge, which may limit the effectiveness of clinical decision-making. Targeted educational interventions may help address these knowledge gaps and potentially improve evidence-based HUA management.

## Introduction

Hyperuricemia (HUA) represents a metabolic condition defined by elevated serum uric acid concentrations beyond 7.0 mg/dL (416.0 μmol/L) in men and 6.0 mg/dL (357.0 μmol/L) in women, constituting a growing global health challenge with rising prevalence rates (1). Chinese epidemiological evidence demonstrates an alarming escalation pattern, with HUA occurrence increasing from 11.1% during 2015–16 to 14.0% in 2018–19, impacting roughly 19.19 million people (2). This metabolic disorder is the primary pathophysiological basis for gout, the most common inflammatory arthritis worldwide, with approximately 41.2 million adults affected globally (3, 4). Furthermore, elevated serum uric acid levels have been associated with numerous comorbidities including chronic kidney disease, cardiovascular events, metabolic syndrome, and increased all-cause mortality, highlighting its multisystemic impact (5). Beyond gout, HUA is frequently encountered in routine internal medicine practice because its clusters with common chronic conditions, such as hypertension, diabetes, obesity, and chronic kidney disease, and may influence long-term cardiometabolic and renal risk stratification and management decisions (6).

Despite its substantial disease burden and established comorbidity associations, HUA remains inadequately addressed in clinical practice. Research indicates significant gaps in disease management, with urate-lowering therapy (ULT) being considerably underutilized among diagnosed patients (7). Moreover, a clear disparity exists between specialty rheumatology guidelines and primary care approaches to HUA management, potentially affecting standardization of care and patient outcomes (8). In many health systems, HUA care is fragmented across specialties and primary care, and the threshold for initiating ULT as well as the intensity of treat-to-target monitoring can vary by clinician training and guideline familiarity. Internal medicine physicians play a pivotal role in HUA management in China, often serving as the primary healthcare providers responsible for the diagnosis and management of the condition and its associated comorbidities. Compared with rheumatologists (who more often manage established gout and advanced urate-lowering strategies) or nephrologists (who may focus on CKD-related urate issues), internal medicine physicians commonly encounter HUA in patients presenting for multimorbidity care and may determine whether HUA is recognized, counseled, monitored, and referred. In contrast to general practitioners, many Chinese internal medicine physicians practice in hospital-based settings and frequently manage complex inpatients and outpatients with cardiometabolic and renal comorbidities, making their KAP particularly relevant for standardizing care pathways. Given the rising prevalence, substantial health burden, and identified risk factors of HUA in the Chinese population—including urbanization, geographic distribution, dietary habits, and comorbid conditions (2)—understanding internal medicine physician’s current knowledge status is crucial for optimizing clinical decision-making and improving standardization of care.

Knowledge, Attitude, and Practice (KAP) surveys serve as valuable diagnostic tools for assessing healthcare providers’ understanding, beliefs, and actions regarding specific medical conditions (9, 10). A recent cross-sectional study among healthcare workers in Shandong province identified overall positive attitudes toward HUA management, yet revealed knowledge gaps regarding serum uric acid thresholds and therapeutic approaches (9). Similar KAP studies from India and Malaysia also reported variability in physicians’ knowledge, perceived barriers, and practice patterns in HUA management (10, 11). Internationally, evidence from high-income settings has likewise shown that gout/HUA care remains suboptimal, with gaps in primary care providers’ knowledge of urate-lowering therapy, treat-to-target monitoring, and guideline-concordant long-term management (12–14). Together, these findings suggest that HUA-related KAP gaps may reflect both individual educational needs and broader differences in clinical training, guideline dissemination, and healthcare-system support. However, comprehensive data on internal medicine physicians’ KAP regarding HUA across broader regions of China and how these dimensions relate to one another remain limited. Specifically, it is still unclear whether internal medicine physicians’ knowledge deficits are consistent across geographically diverse institutions, whether training and clinical exposure are associated with better knowledge and practice, and to what extent knowledge and attitudes translate into self-reported clinical practices. The lack of large-scale, multicenter studies examining internal medicine physicians’ knowledge and practices regarding HUA in China represents a significant research gap. Therefore, this study aimed (1) to describe the levels of KAP regarding HUA among internal medicine physicians across multiple regions of China; (2) to identify participant and practice characteristics associated with KAP scores; and (3) to examine the relationships among KAP, including whether attitude mediates the association between knowledge and practice. Based on the KAP framework, we hypothesized that higher knowledge and more positive attitudes would be associated with better self-reported practices.

## Materials and methods

### Study design and participants

We conducted this observational cross-sectional investigation from November 2024 through March 2025 across diverse healthcare institutions. Twenty-five hospitals contributed to this research, representing broad geographical coverage throughout China, encompassing areas including Chongqing, Beijing, Xinjiang, Sichuan, Guizhou, Guangxi, Qinghai, Wuhan, Tibet, Shanxi, and Fujian. The target population comprised licensed physicians currently working in internal medicine departments. Eligibility was defined based on current employment in internal medicine departments.

The study was approved by the Ethics Committee of the Children’s Hospital of Chongqing Medical University. All participants were informed about the study protocol and provided informed consent to participate. All methods were performed in accordance with relevant guidelines. All procedures were performed in accordance with the ethical standards laid down in the 1964 Declaration of Helsinki and its later amendments, and informed consent was obtained from all participants prior to data collection. The study did not employ any predefined exclusion criteria, allowing for the broad participation of eligible respondents.

### Questionnaire

Survey instrument construction drew upon established research findings (9) and relevant clinical guidelines, including the *Guideline for the diagnosis and management of hyperuricemia and gout in China (2019)* (15) and the *China multi-disciplinary expert consensus on diagnosis and treatment of hyperuricemia and related diseases (2023 edition)* (16). Questionnaire items were drafted to cover core constructs of the KAP framework, including diagnostic thresholds, complications, pharmacologic and non-pharmacologic management, and routine clinical behaviors.

Prior to formal data collection, a pilot study involving 35 participants was conducted to assess the instrument’s reliability. Reliability testing indicated the overall Cronbach’s alpha coefficient reaching 0.922, suggesting strong internal consistency. During the pilot study, participants were encouraged to provide feedback on any items they found confusing or unclear, and no issue were reported. Minor wording adjustments were made for clarity based on pilot feedback before final deployment.

The finalized version of the questionnaire (Supplementary File), administered in Chinese, was structured into four main sections: demographic information, knowledge, attitudes, and practices. The demographic section collected data on variables including age, gender, education level, income, professional title, years of clinical experience, department, hospital tier, whether the hospital had a teaching or research designation, history of HUA-related training within the past year, the number of HUA patients managed in the previous year, and the respondent’s personal disease history. The knowledge section comprised 12 items, each scored as 1 for a correct response and 0 for an incorrect or uncertain answer, yielding a possible score range from 0 to 12. The attitude section included 9 items measured on a five-point Likert scale ranging from “strongly agree” to “strongly disagree,” with scores from 5 to 1, resulting in a total score range of 9–45. The practice section contained 10 items, also rated on a five-point scale from “never” to “always,” with scores assigned from 1 to 5, and an overall range of 10–50. KAP scores were classified according to Bloom’s cut-off criteria (17, 18). Because the KAP domains differ in scale and measurement properties, we applied Bloom’s cut-offs as a pragmatic, descriptive approach based on the percentage of the maximum possible score (POMP) within each domain (i.e., score/maximum × 100). Consistent with prior KAP research (19–22), POMP thresholds of 80–100%, 60–79%, and <60% were used to categorize good, moderate, and poor performance, respectively. These categories were used for descriptive reporting rather than as psychometrically validated proficiency standards. We adopted these thresholds to facilitate comparability with prior KAP studies in health and clinical populations and to provide an interpretable, standardized summary across domains with different score ranges.

### Questionnaire distribution and quality control

Hospitals were selected using a convenience sampling approach. The 25 participating healthcare facilities were included pragmatically based on feasibility, willingness to participate, and the availability of departmental or professional communication channels for survey distribution. Physicians were also recruited by convenience sampling. The electronic survey link was disseminated through departmental and professional networks, and eligible physicians voluntarily completed the questionnaire after reading the study information. Based on IP-address information, the respondents were mainly distributed across Chongqing, Sichuan, Beijing, Xinjiang, Yunnan, Guizhou, Guangxi, Anhui, Shanxi, Hubei, Shaanxi, and other provinces. The questionnaire was administered electronically using the Sojump website (Wenjuanxing; https://www.wjx.cn/), an online survey platform widely used for academic and institutional surveys. To maintain the integrity of the data and enhance the reliability of the findings, several quality control measures were implemented to identify and exclude invalid or potentially biased responses. First, a general knowledge verification item (“The capital of China is Shanghai”) was embedded within the questionnaire to detect inattentive or careless respondents. Second, any submission completed in less than 90 s was excluded, as this duration was deemed insufficient to allow for careful reading and considered responses. Third, extreme or implausible demographic values, such as an age of 150 years, were identified as outliers and removed to minimize systematic errors and reduce data distortion. Finally, questionnaires displaying uniform or repetitive answer patterns—such as selecting the same option for all items—were excluded, as these were indicative of disengagement or non-serious participation.

### Sample size

The sample size was estimated based on the assumption that 50% of internal medicine physicians would have adequate knowledge of HUA, with a 95% confidence level and a margin of error of 5%. Using the formula for cross-sectional surveys (23),


$$n=\frac{z^{2}\mathrm{pq}}{e^{2}}$$

A minimum of 384 participants was required.

### Statistical analysis

Statistical analyses were employed using STATA version 17.0 (StataCorp, College Station, TX, USA). Continuous data are presented as means with standard deviations (SD). The distribution of scores for each dimension of KAP scores was assessed for normality. The continuous variables that adhered to a normal distribution were analyzed using Student’s *t*-test or analysis of variance (ANOVA). In contrast, those variables exhibiting a skewed distribution were analyzed utilizing the Mann–Whitney *U*-test or the Kruskal-Wallis analysis. Categorical variables were presented as frequencies (*n*) and percentages (%). Spearman’s rank correlation coefficient was used to evaluate the associations among the scores of the three KAP dimensions. In accordance with the KAP theoretical framework, structural equation modeling (SEM) was performed to examine whether attitude mediated the relationship between knowledge and practice. Both direct and indirect effects were estimated and compared. The SEM specified paths from knowledge to attitude and practice and from attitude to practice, reflecting the conceptual KAP sequence. Model estimation used standardized coefficients (*β*). Multivariable regression models were fitted prior to SEM to identify factors associated with each KAP domain and to address potential confounding. KAP scores were treated as continuous outcomes and analyzed using multivariable linear regression, reporting *β* coefficients with 95% confidence intervals (CI). A two-sided *p*-value less than 0.05 was considered statistically significant.

## Results

### Basic information on the population

Data collection yielded 405 initial survey submissions. Subsequent quality screening eliminated 7 responses due to insufficient completion time (<90 s) and 8 responses with incorrect verification answers. A total of 390 valid responses were included in the final analysis, representing an effective response rate of 96.30%. The study comprised 390 participants (84.62% female; mean age 36.02 ± 6.91 years), predominantly working in tertiary (87.44%) and teaching hospitals (89.74%), with 81.79% holding associate/bachelor’s degrees. Detailed baseline characteristics of the study population are summarized in Table 1.

**Table 1:** Baseline characteristics.

*N* = 390	*N* (%)	Knowledge score		Attitude score		Practice score	
		Mean ± SD	*p*	Mean ± SD	*p*	Mean ± SD	*p*
Total score		5.51 ± 2.04		41.67 ± 3.59		40.98 ± 8.45	
Gender			0.001		0.063		0.618
Male	60 (15.38)	6.31 ± 1.95		40.98 ± 3.72		41.83 ± 7.46	
Female	330 (84.62)	5.36 ± 2.02		41.8 ± 3.55		40.83 ± 8.62	
Age (years)	36.02 ± 6.91		0.140		0.123		0.306
<35	145 (37.18)	5.56 ± 2.00		41.16 ± 3.82		41.06 ± 7.83	
35~45	209 (53.59)	5.37 ± 2.11		42.08 ± 3.31		40.62 ± 8.92	
≥45	36 (9.23)	6.08 ± 1.59		41.36 ± 3.96		42.75 ± 7.97	
Education			0.052		0.429		0.399
Associate/Bachelor’s degree	319 (81.79)	5.38 ± 2.08		41.66 ± 3.70		40.81 ± 8.80	
Master’s degree	61 (15.64)	6.04 ± 1.76		41.55 ± 3.11		41.31 ± 6.62	
Doctorate (Ph.D.) and other	10 (2.56)	6.2 ± 1.47		42.8 ± 2.52		44.5 ± 6.50	
Monthly income			0.411		0.876		0.326
<5,000	71 (18.21)	5.67 ± 2.08		41.23 ± 4.09		42.42 ± 7.45	
5,000–10,000	216 (55.38)	5.55 ± 2.03		41.67 ± 3.57		40.77 ± 8.48	
>10,000	103 (26.41)	5.31 ± 2.01		41.98 ± 3.22		40.43 ± 8.98	
Professional title			0.200		0.133		0.151
Junior title or below	119 (30.51)	5.57 ± 2.06		41.13 ± 3.87		40.80 ± 7.55	
Intermediate title	223 (57.18)	5.39 ± 2.05		41.81 ± 3.47		40.57 ± 9.21	
Senior title (including associate senior and full senior)	48 (12.31)	5.89 ± 1.87		42.37 ± 3.23		43.31 ± 6.35	
Years of work experience			0.005		0.073		0.776
≤5 years	63 (16.15)	5.95 ± 1.77		41 ± 3.63		41.60 ± 6.79	
5–10 years	82 (21.03)	5.45 ± 2.08		41.08 ± 3.90		40.74 ± 7.92	
11–15 years	156 (40)	5.11 ± 2.16		42.17 ± 3.27		40.56 ± 9.18	
≥16 years	89 (22.82)	5.95 ± 1.80		41.80 ± 3.69		41.50 ± 8.71	
Department			0.192		0.275		0.445
Department of Nephrology	72 (18.46)	5.84 ± 1.93		42.22 ± 3.06		42.13 ± 6.82	
Other	318 (81.54)	5.43 ± 2.05		41.55 ± 3.69		40.72 ± 8.76	
Hospital grade level			0.240		0.196		0.891
Tertiary hospital	341 (87.44)	5.46 ± 2.01		41.77 ± 3.53		40.94 ± 8.49	
Secondary/Primary hospital	49 (12.56)	5.81 ± 2.21		40.97 ± 3.89		41.26 ± 8.18	
Teaching hospital			0.070		0.089		0.322
Yes	350 (89.74)	5.45 ± 2.02		41.76 ± 3.57		41.12 ± 8.37	
No	40 (10.26)	6.05 ± 2.09		40.85 ± 3.67		39.72 ± 9.13	
Research hospital			0.083		0.271		0.846
Yes	308 (78.97)	5.42 ± 2.00		41.81 ± 3.49		41.00 ± 8.31	
No	82 (21.03)	5.84 ± 2.12		41.14 ± 3.88		40.89 ± 8.98	
Pediatric hospital			0.002		0.239		0.366
Yes	153 (39.23)	5.09 ± 2.02		42 ± 3.38		40.37 ± 9.01	
No	237 (60.77)	5.78 ± 2.00		41.46 ± 3.71		41.37 ± 8.06	
Educational lectures related to hyperuricemia			0.023		0.929		0.001
Yes	88 (22.56)	6.05 ± 1.75		41.54 ± 3.76		43.48 ± 7.02	
No	302 (77.44)	5.35 ± 2.08		41.71 ± 3.54		40.25 ± 8.69	
Number of patients with HUA treated per month			<0.001		0.481		0.101
0	209 (53.59)	5.08 ± 2.17		41.69 ± 3.63		39.77 ± 9.59	
1–5	113 (28.97)	5.69 ± 1.84		41.56 ± 3.47		42.27 ± 6.95	
6–20	46 (11.79)	6.63 ± 1.52		41.43 ± 3.79		41.67 ± 6.28	
20 and more	22 (5.64)	6.27 ± 1.35		42.5 ± 3.39		44.36 ± 5.55	
Patients with HUA in your department			0.002		0.868		0.284
Yes	204 (52.31)	5.85 ± 1.82		41.71 ± 3.49		41.76 ± 7.29	
No	186 (47.69)	5.13 ± 2.19		41.62 ± 3.69		40.12 ± 9.50	
HUA			0.097		0.607		0.195
Yes	28 (7.18)	6.25 ± 1.75		41.21 ± 4.20		39.57 ± 7.76	
No	362 (92.82)	5.45 ± 2.04		41.70 ± 3.54		41.09 ± 8.50	

Data are presented as *n* (%) or mean ± SD. Knowledge, attitude, and practice scores ranged from 0 to 12, 9 to 45, and 10 to 50, respectively. *p* values were calculated using Student’s *t*-test or one-way ANOVA for normally distributed variables and the Mann–Whitney U test or Kruskal–Wallis test for non-normally distributed variables, as appropriate. HUA, hyperuricemia; SD, standard deviation.

Their mean knowledge, attitude, and practice scores were 5.51 ± 2.04 (possible range: 0–12), 41.67 ± 3.59 (possible range: 9–45), and 40.98 ± 8.45 (possible range: 10–50), respectively. To improve interpretability, we additionally report the proportions of participants classified as poor, moderate, and good in each domain using POMP-based Bloom’s cutoffs. According to Bloom’s classification thresholds, 91.54% of participants exhibited inadequate knowledge levels (below 60% of maximum score), whereas most subjects (85.38%) displayed favorable attitudes (exceeding 80% of maximum score). For practice, 30.77% had poor practices, 47.95% moderate practices, and 21.28% showed good practices. Subgroup comparisons of KAP scores across participant and institutional characteristics are presented in Table 1. Males exhibited significantly higher knowledge scores than females (6.31 ± 1.95 vs. 5.36 ± 2.02, *p* = 0.001). Knowledge scores were higher among physicians who regularly managed patients with HUA compared to those without relevant clinical experience (*p* < 0.001). Engagement in HUA-related educational lectures correlated with enhanced knowledge (6.05 ± 1.75 vs. 5.35 ± 2.08, *p* = 0.023) and practice scores (43.48 ± 7.02 vs. 40.25 ± 8.69, *p* = 0.001). No significant associations were observed between KAP outcomes and age, income, department, or hospital level (all *p* > 0.05) (Table 1). In addition, comparisons by professional title, years of practice, hospital type (e.g., general vs. specialty), and teaching status are also displayed in Table 1, showing no statistically differences in overall KAP scores unless otherwise indicated.

### Knowledge, attitude, and practice

Analysis of knowledge domain performance revealed that questions with poorest response accuracy included: “Allopurinol and benzbromarone reduce uric acid levels by inhibiting xanthine oxidase activity, thereby decreasing uric acid synthesis” (K7), which had an accuracy rate of 5.64%; “The target for controlling gout is to maintain serum uric acid levels below 180 μmol/L in the long term” (K5), with 22.56%; and “Serum uric acid levels exceeding 420 μmol/L should be immediately treated with uric acid-lowering medications” (K4), with 34.62% accuracy (Table 2).

**Table 2:** Distribution of knowledge dimension responses.

Knowledge items	Accuracy rate, *n* (%)
1. The current diagnostic criterion for HUA in China refers to a fasting serum uric acid level exceeding 420 μmol/L on two different days, regardless of gender, under a normal purine diet.	213 (54.62)
2. When serum uric acid exceeds the normal concentration, monosodium urate crystals precipitate and deposit, triggering an inflammatory response, releasing pro-inflammatory factors, and causing tissue damage.	311 (79.74)
3. HUA can lead to gout and uric acid nephropathy, and is also an independent risk factor for chronic kidney disease, hypertension, cardiovascular and cerebrovascular diseases, and diabetes.	339 (86.92)
4. Serum uric acid levels exceeding 420 μmol/L should be immediately treated with uric acid-lowering medications.	135 (34.62)
5. The target for controlling gout is to maintain serum uric acid levels below 180 μmol/L in the long term.	88 (22.56)
6. Liver function, renal function, and electrolyte tests must be completed before prescribing medications to patients with HUA.	339 (86.92)
7. Allopurinol and benzbromarone reduce uric acid levels by inhibiting xanthine oxidase activity, thereby decreasing uric acid synthesis.	22 (5.64)
8. Which of the following are first-line medications for treating acute gout attacks?	
Ibuprofen	219 (56.15)
Febuxostat	198 (50.77)
Benzbromarone	223 (57.18)
Colchicine	271 (69.49)
Diclofenac sodium	213 (54.62)

Data are presented as *n* (%). Percentages indicate the proportion of participants who answered each knowledge item correctly. For multiple-choice items, each option was evaluated separately. HUA, hyperuricemia.

Responses to the attitude dimension showed that 52.82% strongly agreed and 36.15% agreed that current patient education and publicity efforts regarding HUA are insufficient (A8). Meanwhile, 86.92% are willing or very willing to participate in further training related to HUA (A9) (Table 3). Responses to the practice dimension showed that 15.64% rarely and 7.18% never remind people around them to pay attention to their diet to prevent the onset of HUA (P9), 7.69% rarely and 6.67% never recommend uric acid-lowering medications for patients with poor self-management or dietary habits (P5), 6.41% rarely and 4.36% never provide individualized dietary recommendations for different patients with HUA (P4) (Table 4).

**Table 3:** Distribution of attitude dimension responses.

Attitude items, *n* (%)	Strongly agree	Agree	Neutral	Disagree	Strongly disagree
1. I believe that the treatment of HUA should be taken seriously.	310 (79.49)	76 (19.49)	3 (0.77)	1 (0.26)	/
2. I believe that patient self-management plays an important role in the treatment of HUA.	313 (80.26)	70 (17.95)	7 (1.79)	/	/
3. I believe that lifestyle changes are important for controlling HUA.	320 (82.05)	66 (16.92)	4 (1.03)	/	/
4. I believe that patients with HUA should undergo regular medical check-ups.	299 (76.67)	89 (22.82)	2 (0.51)	/	/
5. I believe that appropriate medication is key to controlling HUA.	244 (62.56)	107 (27.44)	24 (6.15)	13 (3.33)	2 (0.51)
6. I believe that healthcare professionals play an important role in the treatment of HUA.	238 (61.03)	130 (33.33)	21 (5.38)	1 (0.26)	/
7. I believe that patient education is necessary for the management of HUA.	298 (76.41)	88 (22.56)	4 (1.03)	/	/
8. I believe that current patient education and publicity efforts regarding HUA are insufficient.	206 (52.82)	141 (36.15)	38 (9.74)	4 (1.03)	1 (0.26)
9. I am willing to participate in further training related to HUA.	197 (50.51)	142 (36.41)	43 (11.03)	5 (1.28)	3 (0.77)

Data are presented as *n* (%). Attitude items were scored on a five-point Likert scale from 1 to 5, with higher scores indicating more positive attitudes. The total attitude score ranged from 9 to 45. “/” indicates zero responses. HUA, hyperuricemia.

**Table 4:** Distribution of practice dimension responses.

Practice items, *n* (%)	Always	Often	Sometimes	Occasionally	Never
1. I remind patients with HUA to have regular follow-up check-ups.	209 (53.59)	106 (27.18)	54 (13.85)	12 (3.08)	9 (2.31)
2. In patient education, I emphasize the importance of lifestyle changes.	262 (67.18)	87 (22.31)	32 (8.21)	4 (1.03)	5 (1.28)
3. In clinical practice, I always inquire about patients’ dietary habits.	219 (56.15)	107 (27.44)	47 (12.05)	11 (2.82)	6 (1.54)
4. I provide individualized dietary recommendations for different patients with HUA.	183 (46.92)	100 (25.64)	65 (16.67)	25 (6.41)	17 (4.36)
5. For patients with poor self-management or dietary habits, I recommend uric acid-lowering medications.	149 (38.21)	102 (26.15)	83 (21.28)	30 (7.69)	26 (6.67)
6. During clinical diagnosis and treatment, I actively pay attention to patients’ serum uric acid test results.	181 (46.41)	112 (28.72)	62 (15.9)	23 (5.9)	12 (3.08)
7. For patients with poorly controlled serum uric acid, I actively explore possible causes.	168 (43.08)	100 (25.64)	87 (22.31)	19 (4.87)	16 (4.1)
8. For patients who have already experienced gout attacks, I provide additional patient education to emphasize the importance of treatment and the potential for serious complications.	191 (48.97)	109 (27.95)	61 (15.64)	16 (4.1)	13 (3.33)
9. I regularly study and consult the latest treatment guidelines for HUA.	126 (32.31)	70 (17.95)	105 (26.92)	61 (15.64)	28 (7.18)
10. I remind people around me to pay attention to their diet to prevent the onset of HUA.	200 (51.28)	108 (27.69)	61 (15.64)	14 (3.59)	7 (1.79)

Data are presented as *n* (%). Practice items were scored on a five-point Likert scale from 1 to 5, with higher scores indicating more frequent or better self-reported practices. The total practice score ranged from 10 to 50. HUA, hyperuricemia.

### Correlation analysis

Further correlation analysis revealed positive correlations between knowledge scores and practice scores (*r* = 0.1492, *p* = 0.0031). Additionally, attitude scores were positively correlated with practice scores (*r* = 0.5369, *p* < 0.001) (Supplementary Table S1).

### Multivariable regression

In the multivariable analysis, managing 6–20 HUA patients per month was independently associated with higher knowledge scores (*β* = 1.174, 95% CI: 0.478 to 1.870, *p* = 0.001), whereas working in a children’s specialty hospital was negatively associated with knowledge scores (*β* = −0.586, 95% CI: −0.997 to −0.174, *p* = 0.006). No other variables showed statistically significant associations (Supplementary Table S2). Besides, none of the included variables were significantly associated with attitude scores (all *p* > 0.05) (Supplementary Table S3). And knowledge score (*β* = 0.716, 95% CI: 0.349 to 1.083, *p* < 0.001) and attitude score (*β* = 1.064, 95% CI: 0.863 to 1.264, *p* < 0.001) were independently associated with higher practice scores. In addition, participation in HUA training/lectures was significantly associated with higher practice scores (*β* = 2.346, 95% CI: 0.503 to 4.190, *p* = 0.013) (Supplementary Table S4). Additionally, the results of multivariable analyses indicated that gender, age, and hospital characteristics were not significantly associated with KAP scores.

### SEM analysis

**Figure 1:**
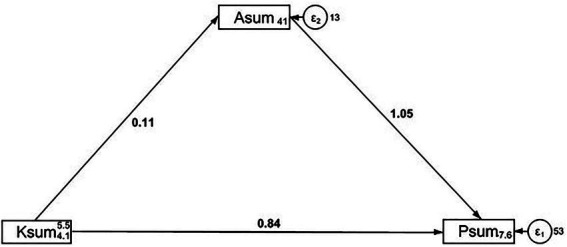
SEM analysis.

Structural equation modeling revealed significant direct pathways from knowledge to practice (*β* = 0.84, 95% CI: 0.48–1.19, *p* < 0.001) and from attitude to practice (*β* = 1.05, CI: 0.85–1.25, *p* < 0.001). However, neither the direct effect of knowledge on attitude nor the indirect effect on practice was statistically significant (Table 5, Figure 1).

**Table 5:** Analysis of direct and indirect effects.

Model paths	Total effects		Direct effect		Indirect effect	
	*β* (95% CI)	*p*	*β* (95% CI)	*p*	*β* (95% CI)	*p*
Asum ←						
Ksum	0.11 (−0.06,0.28)	0.205	0.11 (−0.06,0.28)	0.205		
Psum ←						
Asum	1.05 (0.85,1.25)	<0.001	1.05 (0.85,1.25)	<0.001		
Ksum	0.95 (0.55,1.35)	<0.001	0.84 (0.48,1.19)	<0.001	0.11 (−0.06,0.30)	0.209

Ksum, total knowledge score; Asum, total attitude score; Psum, total practice score; CI, confidence interval. *β* represents the path coefficient estimated in the structural equation model. The model was just-identified; therefore, global fit indices were not interpreted.

## Discussion

Despite internal medicine practitioners showing largely favorable attitudes and acceptable practice patterns regarding HUA care, their fundamental understanding remained insufficient, with knowledge showing no significant correlation with attitudinal measures. Targeted educational interventions are warranted to enhance physicians’ knowledge of HUA, which may further reinforce evidence-based clinical practices.

This pattern—favorable attitudes coexisting with weak knowledge—may reflect several contextual factors. First, HUA is often a chronic, frequently asymptomatic condition; in routine internal medicine practice it may be deprioritized compared with acute presentations or other high-burden chronic diseases, particularly when institutional protocols do not explicitly embed HUA management into workflows (24, 25). Second, guideline exposure may be limited or inconsistent across hospitals and departments, and HUA may receive insufficient emphasis during residency, postgraduate training, and continuing medical education. Third, our poorest-performing knowledge items clustered around pharmacologic mechanisms and treatment targets/thresholds, such as urate-lowering drug mechanisms and target urate levels, suggesting that education may be fragmented and that misconceptions persist in areas most relevant to prescribing decisions. Similar patterns of inconsistent clinical knowledge have been observed in other studies involving non-specialist healthcare providers (26, 27). Collectively, these factors may explain why physicians can endorse HUA management in principle yet lack the detailed, guideline-consistent knowledge needed for standardized decision-making.

A further consideration is that attitude and practice were self-reported and therefore susceptible to social desirability bias and self-valorization, particularly for items framed around “should” behaviors (e.g., guideline consultation, counseling, and adherence to recommended management). In this context, the observed high prevalence of positive attitudes (86.92%) may partly reflect respondents’ tendency to select socially acceptable responses rather than their true beliefs or routine behaviors. This potential inflation is supported by the common KAP pattern in which attitudinal endorsement exceeds demonstrated knowledge and may not fully align with objective practice. Accordingly, the attitude and practice findings should be interpreted as physicians stated orientation and self-perceived behaviors, not as direct evidence of actual clinical performance.

Aligning with prior KAP research involving healthcare personnel in Shandong Province, China (9), our findings similarly identified inadequate knowledge levels alongside largely positive attitudes concerning HUA care, underscoring pervasive educational deficits and necessitating enhanced guideline distribution across the nation. However, our study showed a moderate level of practice, whereas the Shandong study reported more proactive practices. This discrepancy may reflect differences in institutional emphasis, local training programs, or clinical exposure. Notably, a study from India reported that the majority of physicians demonstrated adequate knowledge and adherence to best practices in HUA management (10). This contrast may be attributed to differences in study populations: the Indian study focused on physicians managing patients with comorbid conditions such as hypertension, diabetes, and metabolic syndrome, while our study targeted general internal medicine physicians, many of whom had limited direct experience in treating HUA. This interpretation is consistent with our univariate and multivariable results indicating that greater clinical exposure (e.g., managing more HUA patients per month) and participation in HUA training/lectures were associated with improved knowledge and/or practice.

The present study reveals several challenges in translating physicians’ knowledge into clinical practice, despite generally positive attitudes. This disconnect is not uncommon in the management of chronic, asymptomatic conditions such as HUA, where clinical action may be deprioritized in the absence of acute symptoms or clear institutional protocols (24, 25). In addition to individual knowledge gaps, structural barriers may contribute to the attitude–practice gap, including limited consultation time for lifestyle counseling, lack of standardized departmental pathways, absence of decision-support tools, competing clinical priorities, and limited opportunities for continuing education. Comparable findings have been reported in other chronic disease areas, where provider knowledge did not always translate into consistent clinical behavior (28, 29). Our SEM results further confirmed that both knowledge and attitude had significant direct effects on practice, but attitude did not mediate the relationship between knowledge and practice. This finding emphasizes the need for practical reinforcement mechanisms—such as clinical decision tools, structured guidelines, and institutional training—to bridge the gap between what physicians know and what they do in practice (30, 31).

Notably, several items with low correct response rates involved pharmacologic mechanisms, treatment thresholds, and dietary guidance, which are critical for effective HUA management. These findings underscore the need for more tailored, clinically focused education programs that address practical issues physicians encounter in daily practice. Prior research has highlighted the importance of targeted clinical training to correct knowledge gaps, such as the misunderstanding of urate-lowering medication mechanisms and recommended uric acid targets (32, 33). Therefore, training programs should prioritize guideline-consistent urate targets and monitoring, evidence-based indications and timing for initiating urate-lowering therapy, key pharmacologic principles and common misconceptions, and efficient, feasible dietary/lifestyle counseling messages that can be delivered in routine practice.

Although most physicians demonstrated positive attitudes, there remained a gap in routine clinical behaviors, such as guideline consultation and patient dietary counseling. This suggests that attitudinal positivity alone does not guarantee high-quality care. Interventions such as continuous professional development programs, case-based training, and integration of HUA care into chronic disease pathways may help standardize physician behaviors over time (34). In addition, community-based participatory education and peer-led models have been shown to improve adherence in similar health domains, and could be explored as feasible, resource-efficient approaches in regions with limited healthcare staffing (35).

In light of these findings, several feasible recommendations can be proposed. First, targeted continuing medical education programs should be developed to address clinical misconceptions and standardize knowledge of HUA diagnostic and treatment thresholds. Second, internal medicine departments should embed guideline-based HUA management into daily practice workflows, for example, quick-reference algorithms, standardized counseling templates, and structured follow-up reminders. Third, professional societies may consider integrating HUA content into broader chronic disease training curricula and leveraging digital platforms for periodic reinforcement (36, 37).

### Strengths and limitations

This study has several strengths. It addresses a clinically meaningful topic using a multicenter design and a structured KAP framework to evaluate physicians’ HUA-related KAP. The inclusion of correlation analyses, multivariable regression, and structural equation modeling adds analytical depth by examining relationships among domains and identifying factors associated with KAP outcomes.

Several limitations should be noted. First, the cross-sectional design precludes causal inference; observed associations (e.g., between training and practice) cannot establish directionality. Second, attitude and practice were self-reported, which introduces social desirability and recall bias and may overestimate guideline consultation, counseling frequency, or other “should-do” behaviors. Future work should triangulate self-reports with objective indicators such as prescribing audits, chart reviews, or EMR-based quality metrics. Third, the sample was concentrated in tertiary and teaching hospitals, and the predominance of female respondents may limit generalizability to male physicians and to non-tertiary, community, and non-teaching settings; potential institutional clustering may also affect external validity. Fourth, although the questionnaire was developed and applied within a KAP framework, formal content validity indices and construct validity analyses were not performed. Therefore, further validation of the instrument is warranted. Finally, this study did not assess patient-level treatment processes or clinical outcomes; therefore, whether improving physicians’ KAP can translate into better HUA management and patient outcomes should be evaluated in future longitudinal or interventional studies.

## Conclusion

Inadequate knowledge, coupled with generally positive attitudes and moderate practices, was observed among internal medicine physicians regarding HUA management. Targeted educational interventions are warranted to enhance physicians’ knowledge, which may further reinforce evidence-based practices and improve patient outcomes in HUA care.

## Data Availability

The original contributions presented in the study are included in the article/Supplementary material, further inquiries can be directed to the corresponding author.
