# Supplementary material for: Knowledge, attitudes, and practices regarding hyperuricemia among physicians in internal medicine departments: a multicenter cross-sectional survey in China
Source: Front Public Health. 2026 May 18;14:1750197. doi: 10.3389/fpubh.2026.1750197 (PMC13223091; doi:10.3389/fpubh.2026.1750197)
Supplement: Supplementary file 1 [file Data_Sheet_1.docx]

**Table S1. Correlation analysis**

|  | **Knowledge** | **Attitude** | **Practice** |
| --- | --- | --- | --- |
| **Knowledge** | 1 |  |  |
| **Attitude** | 0.0443 (P=0.383) | 1 |  |
| **Practice** | 0.1492 (P=0.003) | 0.5369 (P < 0.001) | 1 |

Note: Values are Spearman’s rank correlation coefficients, with corresponding P values shown in parentheses.

**Table S2. Univariable and multivariable linear regression of knowledge score**

| Variable | Category (reference) | Univariable β (95% CI) | P | Multivariable β (95% CI) | P |
| --- | --- | --- | --- | --- | --- |
| Gender | Male (ref) | — | — | — | — |
|  | Female | -0.950 (-1.505, -0.395) | 0.001 | -0.454 (-1.013, 0.106) | 0.113 |
| Age (years) | <35 (ref) | — | — | — | — |
|  | 35–45 | -0.188 (-0.620, 0.245) | 0.394 | — | — |
|  | ≥45 | 0.518 (-0.227, 1.262) | 0.172 | — | — |
| Education | College/Undergraduate (ref) | — | — | — | — |
|  | Master’s | 0.661 (0.104, 1.217) | 0.020 | 0.300 (-0.252, 0.851) | 0.287 |
|  | Doctorate/Other | 0.811 (-0.468, 2.090) | 0.213 | 0.314 (-0.975, 1.602) | 0.634 |
| Monthly income | <5000 (ref) | — | — | — | — |
|  | 5000–10000 | -0.121 (-0.669, 0.428) | 0.666 | — | — |
|  | >10000 | -0.365 (-0.984, 0.253) | 0.246 | — | — |
| Professional title | Junior or below (ref) | — | — | — | — |
|  | Intermediate | -0.172 (-0.627, 0.282) | 0.457 | — | — |
|  | Senior (associate senior/senior) | 0.324 (-0.360, 1.009) | 0.352 | — | — |
| Years of practice | ≤5 (ref) | — | — | — | — |
|  | 5–10 | -0.501 (-1.164, 0.161) | 0.138 | -0.388 (-1.042, 0.267) | 0.246 |
|  | 11–15 | -0.837 (-1.427, -0.247) | 0.006 | -0.520 (-1.127, 0.088) | 0.095 |
|  | ≥16 | 0.003 (-0.648, 0.654) | 0.994 | 0.214 (-0.441, 0.870) | 0.522 |
| Department | Nephrology (ref) | — | — | — | — |
|  | Other | -0.410 (-0.932, 0.112) | 0.123 | — | — |
| Hospital tier | Tertiary (ref) | — | — | — | — |
|  | Primary/Secondary | 0.347 (-0.265, 0.959) | 0.265 | — | — |
| Teaching hospital | No (ref) | — | — | — | — |
|  | Yes | -0.599 (-1.266, 0.068) | 0.078 | — | — |
| Research hospital | No (ref) | — | — | — | — |
|  | Yes | -0.416 (-0.913, 0.081) | 0.100 | — | — |
| Children’s specialty hospital | No (ref) | — | — | — | — |
|  | Yes | -0.683 (-1.093, -0.272) | 0.001 | -0.586 (-0.997, -0.174) | 0.006 |
| HUA training/lecture in past year | No (ref) | — | — | — | — |
|  | Yes | 0.703 (0.222, 1.183) | 0.004 | 0.276 (-0.220, 0.772) | 0.276 |
| HUA patients managed/month (past year) | 0 (ref) | — | — | — | — |
|  | 1–5 | 0.613 (0.160, 1.066) | 0.008 | 0.327 (-0.207, 0.861) | 0.231 |
|  | 6–20 | 1.544 (0.912, 2.176) | <0.001 | 1.174 (0.478, 1.870) | 0.001 |
|  | ≥20 | 1.187 (0.317, 2.056) | 0.008 | 0.482 (-0.507, 1.471) | 0.340 |
| HUA patients in department | No (ref) | — | — | — | — |
|  | Yes | 0.713 (0.313, 1.114) | 0.001 | 0.222 (-0.256, 0.700) | 0.364 |
| Self-reported HUA | No (ref) | — | — | — | — |
|  | Yes | 0.794 (0.011, 1.577) | 0.047 | 0.520 (-0.243, 1.282) | 0.183 |

Note: β represents the regression coefficient, and 95% CI represents the 95% confidence interval. The knowledge score ranged from 0 to 12. Reference categories are indicated by “ref.” Variables with “—” were not included in the final multivariable model or were used as reference categories. HUA, hyperuricemia; CI, confidence interval.

**Table S3. Univariable and multivariable linear regression of attitude score**

| Variable | Category (reference) | Univariable β (95% CI) | P | Multivariable β (95% CI) | P |
| --- | --- | --- | --- | --- | --- |
| Knowledge score | Per 1-point increase | 0.113 (-0.062, 0.288) | 0.207 | — | — |
| Gender | Male (ref) | — | — | — | — |
|  | Female | 0.817 (-0.172, 1.805) | 0.105 | — | — |
| Age (years) | <35 (ref) | — | — | — | — |
|  | 35–45 | 0.916 (0.157, 1.675) | 0.018 | 0.208 (-0.940, 1.357) | 0.722 |
|  | ≥45 | 0.196 (-1.112, 1.503) | 0.769 | -0.698 (-2.516, 1.120) | 0.452 |
| Education | College/Undergraduate (ref) | — | — | — | — |
|  | Master’s | -0.104 (-1.092, 0.884) | 0.836 | — | — |
|  | Doctorate/Other | 1.139 (-1.131, 3.408) | 0.325 | — | — |
| Monthly income | <5000 (ref) | — | — | — | — |
|  | 5000–10000 | 0.432 (-0.534, 1.398) | 0.380 | — | — |
|  | >10000 | 0.741 (-0.348, 1.830) | 0.182 | — | — |
| Professional title | Junior or below (ref) | — | — | — | — |
|  | Intermediate | 0.677 (-0.121, 1.476) | 0.096 | 0.182 (-0.884, 1.248) | 0.738 |
|  | Senior (associate senior/senior) | 1.241 (0.038, 2.443) | 0.043 | 0.954 (-0.582, 2.490) | 0.224 |
| Years of practice | ≤5 (ref) | — | — | — | — |
|  | 5–10 | 0.085 (-1.090, 1.261) | 0.887 | -0.032 (-1.297, 1.233) | 0.960 |
|  | 11–15 | 1.180 (0.132, 2.227) | 0.027 | 0.793 (-0.700, 2.285) | 0.299 |
|  | ≥16 | 0.809 (-0.346, 1.964) | 0.169 | 0.528 (-1.183, 2.239) | 0.546 |
| Department | Nephrology (ref) | — | — | — | — |
|  | Other | -0.672 (-1.592, 0.248) | 0.152 | — | — |
| Hospital tier | Tertiary (ref) | — | — | — | — |
|  | Primary/Secondary | -0.795 (-1.871, 0.282) | 0.148 | — | — |
| Teaching hospital | No (ref) | — | — | — | — |
|  | Yes | 0.919 (-0.258, 2.095) | 0.125 | — | — |
| Research hospital | No (ref) | — | — | — | — |
|  | Yes | 0.669 (-0.207, 1.544) | 0.134 | — | — |
| Children’s specialty hospital | No (ref) | — | — | — | — |
|  | Yes | 0.536 (-0.195, 1.267) | 0.150 | — | — |
| HUA training/lecture in past year | No (ref) | — | — | — | — |
|  | Yes | -0.167 (-1.023, 0.690) | 0.702 | — | — |
| HUA patients managed/month (past year) | 0 (ref) | — | — | — | — |
|  | 1–5 | -0.132 (-0.958, 0.694) | 0.753 | — | — |
|  | 6–20 | -0.264 (-1.416, 0.888) | 0.653 | — | — |
|  | ≥20 | 0.801 (-0.784, 2.387) | 0.321 | — | — |
| HUA patients in department | No (ref) | — | — | — | — |
|  | Yes | 0.087 (-0.630, 0.803) | 0.812 | — | — |
| Self-reported HUA | No (ref) | — | — | — | — |
|  | Yes | -0.496 (-1.881, 0.890) | 0.482 | — | — |

Note: β represents the regression coefficient, and 95% CI represents the 95% confidence interval. The knowledge score ranged from 0 to 12. Reference categories are indicated by “ref.” Variables with “—” were not included in the final multivariable model or were used as reference categories. HUA, hyperuricemia; CI, confidence interval.

**Table S4. Univariable and multivariable linear regression of practice score**

| Variable | Category (reference) | Univariable β (95% CI) | P | Multivariable β (95% CI) | P |
| --- | --- | --- | --- | --- | --- |
| Knowledge score | Per 1-point increase | 0.960 (0.558, 1.361) | <0.001 | 0.716 (0.349, 1.083) | <0.001 |
| Attitude score | Per 1-point increase | 1.085 (0.878, 1.293) | <0.001 | 1.064 (0.863, 1.264) | <0.001 |
| Gender | Male (ref) | — | — | — | — |
|  | Female | -1.003 (-3.336, 1.330) | 0.398 | — | — |
| Age (years) | <35 (ref) | — | — | — | — |
|  | 35–45 | -0.435 (-2.231, 1.361) | 0.634 | — | — |
|  | ≥45 | 1.688 (-1.406, 4.782) | 0.284 | — | — |
| Education | College/Undergraduate (ref) | — | — | — | — |
|  | Master’s | 0.500 (-1.823, 2.822) | 0.673 | — | — |
|  | Doctorate/Other | 3.688 (-1.648, 9.024) | 0.175 | — | — |
| Monthly income | <5000 (ref) | — | — | — | — |
|  | 5000–10000 | -1.649 (-3.921, 0.622) | 0.154 | — | — |
|  | >10000 | -1.986 (-4.547, 0.575) | 0.128 | — | — |
| Professional title | Junior or below (ref) | — | — | — | — |
|  | Intermediate | -0.228 (-2.109, 1.653) | 0.812 | — | — |
|  | Senior (associate senior/senior) | 2.506 (-0.327, 5.339) | 0.083 | — | — |
| Years of practice | ≤5 (ref) | — | — | — | — |
|  | 5–10 | -0.859 (-3.650, 1.931) | 0.545 | — | — |
|  | 11–15 | -1.039 (-3.525, 1.447) | 0.412 | — | — |
|  | ≥16 | -0.098 (-2.840, 2.645) | 0.944 | — | — |
| Department | Nephrology (ref) | — | — | — | — |
|  | Other | -1.416 (-3.582, 0.751) | 0.200 | — | — |
| Hospital tier | Tertiary (ref) | — | — | — | — |
|  | Primary/Secondary | 0.321 (-2.221, 2.863) | 0.804 | — | — |
| Teaching hospital | No (ref) | — | — | — | — |
|  | Yes | 1.404 (-1.370, 4.177) | 0.320 | — | — |
| Research hospital | No (ref) | — | — | — | — |
|  | Yes | 0.120 (-1.948, 2.187) | 0.910 | — | — |
| Children’s specialty hospital | No (ref) | — | — | — | — |
|  | Yes | -0.996 (-2.719, 0.726) | 0.256 | — | — |
| HUA training/lecture in past year | No (ref) | — | — | — | — |
|  | Yes | 3.234 (1.244, 5.223) | 0.002 | 2.346 (0.503, 4.190) | 0.013 |
| HUA patients managed/month (past year) | 0 (ref) | — | — | — | — |
|  | 1–5 | 2.494 (0.574, 4.415) | 0.011 | 1.611 (-0.116, 3.337) | 0.068 |
|  | 6–20 | 1.894 (-0.785, 4.573) | 0.165 | 0.477 (-1.936, 2.890) | 0.699 |
|  | ≥20 | 4.584 (0.897, 8.271) | 0.015 | 1.720 (-1.606, 5.046) | 0.312 |
| HUA patients in department | No (ref) | — | — | — | — |
|  | Yes | 1.646 (-0.033, 3.325) | 0.055 | — | — |
| Self-reported HUA | No (ref) | — | — | — | — |
|  | Yes | -1.523 (-4.782, 1.737) | 0.359 | — | — |

Note: β represents the regression coefficient, and 95% CI represents the 95% confidence interval. The knowledge score ranged from 0 to 12. Reference categories are indicated by “ref.” Variables with “—” were not included in the final multivariable model or were used as reference categories. HUA, hyperuricemia; CI, confidence interval.
