# Supplementary material for: Knowledge, attitudes, and practices regarding hyperuricemia among physicians in internal medicine departments: a multicenter cross-sectional survey in China
Source: Front Public Health. 2026 May 18;14:1750197. doi: 10.3389/fpubh.2026.1750197 (PMC13223091; doi:10.3389/fpubh.2026.1750197)
Supplement: Supplementary file 2 [file Supplementary_file_1.docx]

| Questionnaire ID： | | |
| --- | --- | --- |
| Dear Participant,  We are researchers from the hospital and sincerely invite you to participate in our study. This research aims to understand the knowledge, attitudes, and practices of internal medicine physicians regarding hyperuricemia, in order to provide a basis for developing effective intervention strategies. The findings may help more people and improve their health in the future. Your participation is voluntary. This study has been reviewed and approved by the Ethics Review Committee. If you agree to participate, please read the following instructions:  1. Please complete the questionnaire. There are no right or wrong answers. You only need to answer based on your actual situation. If you have any questions during the process, feel free to ask us. After completing the questionnaire, please submit it promptly.  2. This study involves only a simple questionnaire and will not cause any harm to your physical or mental health. However, it includes some personal information such as your gender and age. We will strictly protect your privacy and ensure that your information will not be disclosed. Please feel free to answer.  3. As a participant, you may access relevant information and research progress at any time. If you decide to withdraw from the study, please inform us, and your data will not be included in the research results.  Finally, we sincerely thank you for taking the time out of your busy schedule to support our scientific research!  □I have been informed and agree that the data collected may be used for scientific research.  Informed Consent Signature:  Date of Participation: ________ Year ________ Month ________ Day | | |
| **Part 1 Basic Information** | | |
| 1. **Your gender:** | | a. Male b. Female |
| 1. **Your age: ________ years old.** | | |
| 1. **Your highest educational level:** | | a. Associate/Bachelor's degree b. Master's degree c. Doctorate (Ph.D.) d. Postdoctoral |
| 1. **Your monthly income:** | | a.<2000  b.2000-5000  c.5000-10000  d.10000-20000  e.>20000 |
| 1. **Your professional title:** | | a. No title b. Junior title c. Intermediate title d. Senior title (including associate senior and full senior) |
| 1. **Years of work experience:** | | a. ≤5 years b. 5–10 years c. 11–15 years d. ≥16 years |
| 1. **Your department:** | | a. Department of Rheumatology and Immunology b. Department of Nephrology c. Department of Endocrinology and Metabolism d. Department of Cardiovascular Medicine e. Department of Neurology f. Health Management Center g. Other departments |
| 1. **Grade level of your hospital:** | | a. Tertiary hospital b. Secondary hospital c. Primary hospital d. Private hospital |
| 1. **Is your hospital a teaching hospital?** | | a. Yes b. No |
| 1. **Is your hospital a research hospital?** | | a. Yes b. No |
| 1. **Is your hospital a pediatric hospital?** | | a. Yes b. No |
| 1. **In the past year, have you participated in any training or educational lectures related to hyperuricemia?** | | a. Yes b. No |
| 1. **On average, how many patients with hyperuricemia have you treated per month in the past year?** | | a. 0 b. 1–5 c. 6–20 d. 21–50 e. More than 50 |
| 1. **Are there patients with hyperuricemia in your department?** | | a. Yes b. No |
| 1. **Do you have hyperuricemia yourself?** | | a. Yes b. No |

| **Part 2 Knowledge About Hyperuricemia** | | | |  |
| --- | --- | --- | --- | --- |
| 1. **The current diagnostic criterion for hyperuricemia in China refers to a fasting serum uric acid level exceeding 420 μmol/L on two different days, regardless of gender, under a normal purine diet.** | a. True | b. False | c. Not sure | |
| 1. **When serum uric acid exceeds the normal concentration, monosodium urate crystals precipitate and deposit, triggering an inflammatory response, releasing pro-inflammatory factors, and causing tissue damage.** | a. True | b. False | c. Not sure | |
| 1. **Hyperuricemia can lead to gout and uric acid nephropathy, and is also an independent risk factor for chronic kidney disease, hypertension, cardiovascular and cerebrovascular diseases, and diabetes.** | a. True | b. False | c. Not sure | |
| 1. **Serum uric acid levels exceeding 420 μmol/L should be immediately treated with uric acid-lowering medications.** | a. True | b. False | c. Not sure | |
| 1. **The target for controlling gout is to maintain serum uric acid levels below 180 μmol/L in the long term.** | a. True | b. False | c. Not sure | |
| 1. **Liver function, renal function, and electrolyte tests must be completed before prescribing medications to patients with hyperuricemia.** | a. True | b. False | c. Not sure | |
| 1. **Allopurinol and benzbromarone reduce uric acid levels by inhibiting xanthine oxidase activity, thereby decreasing uric acid synthesis.** | a. True | b. False | c. Not sure | |
| 1. **Which of the following are first-line medications for treating acute gout attacks?** |  |  |  | |
| Ibuprofen | a. True | b. False | c. Not sure | |
| Febuxostat | a. True | b. False | c. Not sure | |
| Benzbromarone | a. True | b. False | c. Not sure | |
| Colchicine | a. True | b. False | c. Not sure | |
| Diclofenac sodium | a. True | b. False | c. Not sure | |

Note: The correct answer is marked in red.

| **Part 3 Attitudes Toward Hyperuricemia** | | | | | |
| --- | --- | --- | --- | --- | --- |
| 1. **I believe that the treatment of hyperuricemia should be taken seriously.** | a. Strongly agree | b. Agree | c. Neutral | d. Disagree | e. Strongly disagree |
| 1. **I believe that patient self-management plays an important role in the treatment of hyperuricemia.** | a. Strongly agree | b. Agree | c. Neutral | d. Disagree | e. Strongly disagree |
| 1. **I believe that lifestyle changes are important for controlling hyperuricemia.** | a. Strongly agree | b. Agree | c. Neutral | d. Disagree | e. Strongly disagree |
| 1. **I believe that patients with hyperuricemia should undergo regular medical check-ups.** | a. Strongly agree | b. Agree | c. Neutral | d. Disagree | e. Strongly disagree |
| 1. **I believe that appropriate medication is key to controlling hyperuricemia.** | a. Strongly agree | b. Agree | c. Neutral | d. Disagree | e. Strongly disagree |
| 1. **I believe that healthcare professionals play an important role in the treatment of hyperuricemia.** | a. Strongly agree | b. Agree | c. Neutral | d. Disagree | e. Strongly disagree |
| 1. **I believe that patient education is necessary for the management of hyperuricemia.** | a. Strongly agree | b. Agree | c. Neutral | d. Disagree | e. Strongly disagree |
| 1. **I believe that current patient education and publicity efforts regarding hyperuricemia are insufficient.** | a. Strongly agree | b. Agree | c. Neutral | d. Disagree | e. Strongly disagree |
| 1. **I am willing to participate in further training related to hyperuricemia.** | a. Strongly agree | b. Agree | c. Neutral | d. Disagree | e. Strongly disagree |

| **Part 4 Practices Related to Hyperuricemia** | | | | | |
| --- | --- | --- | --- | --- | --- |
| 1. **I remind patients with hyperuricemia to have regular follow-up check-ups.** | a. Always | b. Often | c. Sometimes | d. Occasionally | e. Never |
| 1. **In patient education, I emphasize the importance of lifestyle changes.** | a. Always | b. Often | c. Sometimes | d. Occasionally | e. Never |
| 1. **In clinical practice, I always inquire about patients' dietary habits.** | a. Always | b. Often | c. Sometimes | d. Occasionally | e. Never |
| 1. **I provide individualized dietary recommendations for different patients with hyperuricemia.** | a. Always | b. Often | c. Sometimes | d. Occasionally | e. Never |
| 1. **For patients with poor self-management or dietary habits, I recommend uric acid-lowering medications.** | a. Always | b. Often | c. Sometimes | d. Occasionally | e. Never |
| 1. **During clinical diagnosis and treatment, I actively pay attention to patients' serum uric acid test results.** | a. Always | b. Often | c. Sometimes | d. Occasionally | e. Never |
| 1. **For patients with poorly controlled serum uric acid, I actively explore possible causes.** | a. Always | b. Often | c. Sometimes | d. Occasionally | e. Never |
| 1. **For patients who have already experienced gout attacks, I provide additional patient education to emphasize the importance of treatment and the potential for serious complications.** | a. Always | b. Often | c. Sometimes | d. Occasionally | e. Never |
| 1. **I regularly study and consult the latest treatment guidelines for hyperuricemia.** | a. Always | b. Often | c. Sometimes | d. Occasionally | e. Never |
| 1. **I remind people around me to pay attention to their diet to prevent the onset of hyperuricemia.** | a. Always | b. Often | c. Sometimes | d. Occasionally | e. Never |
